# Spontaneous Room-Temperature Solid-State Reaction at the MoS_2_/Ti Interface: Implications for Contact Engineering

**DOI:** 10.1021/acsanm.5c04350

**Published:** 2025-12-05

**Authors:** Bazlul Karim, Luka Pirker, Jan Plšek, Václav Valeš, Martin Vondráček, Michaela Hanušová, Viktor Zólyomi, Michele Gastaldo, Abhilash Bajpai, Jakob Ziewer, Fumin Huang, Jan Honolka, Otakar Frank, Matěj Velický, Martin Kalbáč

**Affiliations:** † J. Heyrovský Institute of Physical Chemistry, 86875 Czech Academy of Sciences, Dolejškova 2155/3, 182 23 Prague 8, Czech Republic; ‡ Institute of Physics, 48311 Czech Academy of Sciences, Na Slovance 1999/2, 182 21 Prague 8, Czech Republic; § Faculty of Chemical Engineering, University of Chemistry and Technology, Prague, Technická 5, 166 28 Prague 6, Czech Republic; ∥ Hartree Centre, 86889 STFC Daresbury Laboratory, Daresbury WA4 4AD, United Kingdom; ⊥ Centre for Quantum Materials and Technologies, School of Mathematics and Physics, Queen’s University Belfast, University Road, Belfast BT7 1NN, United Kingdom

**Keywords:** UHV exfoliation, MoS_2_, titanium, interfacial reaction, XPS, Raman spectroscopy

## Abstract

High-quality interfaces with metals are essential for exploiting two-dimensional materials in nanoscale solid-state devices. Conventional strategies rely on depositing metals with a suitable work function at low kinetic energies to minimize damage. Direct exfoliation of layered materials onto clean metal surfaces under ultrahigh vacuum offers a promising alternative, producing pristine, oxide-free interfaces while avoiding the disorder induced by high-energy metal deposition. TMDCs possess unique properties, including a direct band gap, high carrier mobility, and strong spin–orbit coupling, which makes them highly promising for high-performance electronic and optoelectronic devices such as transistors, photodetectors, and solar cells, as well as for emerging quantum technologies. While the metal-assisted exfoliation is well established for gold and other noble metals, its applicability to more reactive metals remains unclear. To address this question, we exfoliate large-area MoS_2_ layers on titanium under ultrahigh vacuum and characterize the resulting heterostructures using X-ray photoelectron and Raman spectroscopy. The monolayer yield is over 75%, but in contrast to noble metals, Ti reacts with exfoliated MoS_2_ at room temperature, producing metallic molybdenum and various sulfur species upon simple physical contact, and only ∼6% of MoS_2_ remains in its pristine form. The degradation also propagates to the second MoS_2_ layer in the bilayer case. In trilayer MoS_2_, the degradation and subsequent oxidation upon air exposure are significantly reduced, and the top layer remains intact and partially protects the interface. These findings likely apply to other material combinations and suggest opportunities for engineering low-resistance contacts, particularly with reactive and high-melting-point metals.

## Graphical abstract

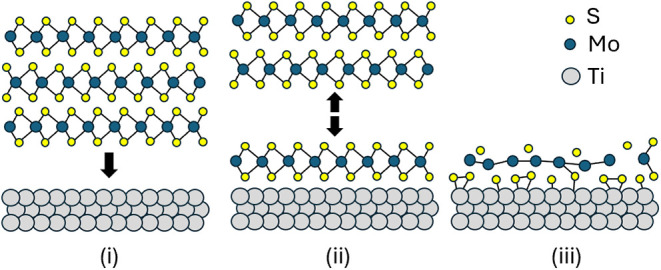

## Introduction

Transition-metal dichalcogenides (TMDCs), especially molybdenum disulfide monolayers, have been extensively investigated as prospective materials for nanoelectronics due to their unique properties, such as a direct band gap, high carrier mobility, strong spin–orbit coupling, valley-selective circular dichroism, large piezoelectricity, extreme mechanical flexibility, and so on.^1–6^ These characteristics make TMDCs highly promising for high-performance electronic devices, such as transistors and photodetectors, energy conversion systems including solar cells and piezoelectric generators, and biomedical technologies for biosensing, imaging, and drug delivery. TMDCs are also being explored for applications in membranes, catalysis, and advanced material synthesis, highlighting their versatility across multiple technological areas.^7–9^ However, the high contact resistance at the MoS_2_/metal junction arising from the Schottky barrier and Fermi-level (*E*_F_) pinning has remained a pressing issue for metals conventionally used as electrical contacts, thus limiting the performance of the nanoscale devices.^10,11^ To address this problem, different approaches have been explored, including the utilization of graphene,^12^ semimetallic bismuth,^13^ and low work-function scandium or titanium contacts.^14^ Titanium is frequently considered because of its small lattice-mismatch with monolayer MoS_2_ (∼1%) and higher density of delocalized states at *E*_F_ at the interface, compared to Au.^15^ In addition, deposited Ti gives rise to a van der Waals gap between the Ti and MoS_2_,^16^ which can alleviate the Fermi-level pinning at the metal–semiconductor interface.^17,18^

Several different metals have been used to mechanically exfoliate MoS_2_ and other TMDCs.^19–26^ The metal-assisted exfoliation yields large-area monolayers (1L) owing to the TMDC-metal binding energy, interfacial strain, and hybridization effects, which weaken the adhesion between the two layers nearest to the interface.^21,26–28^ On the other hand, the metallic surface must remain unoxidized and free from airborne contamination,^21,26,29^ which would otherwise weaken its interaction with the TMDC and drastically reduce the exfoliation yield. Consequently, gold remains the frontrunner in terms of both the exfoliated area and monolayer yield under ambient conditions, primarily due to its oxidation resistance. Recent reports of metal-assisted exfoliation under ultrahigh vacuum (UHV), which offers an oxygen- and contaminant-free environment, achieved large exfoliation yields approaching 100% also on non-Au metals.^26,30–32^ Consequently, UHV enables the production of MoS_2_ monolayers directly on metals with properties suitable for electrical contacts, such as titanium, which has recently been predicted to be a strong candidate for mechanical exfoliation due to its high adhesion energy with TMDCs,^33^ but which readily oxidizes in air. Despite their relevance in device architectures, including the favorable interfacial properties of Ti and the extensive characterization of MoS_2_ as a model TMDC, clean and structurally defined MoS_2_-Ti interfaces have not yet been realized, which motivates our focus on this material system.

To address the challenges mentioned above, we exfoliate MoS_2_ on Ti under UHV and investigate the interactions between the two materials. We assess the impact of Ti on different physical properties of the MoS_2_ monolayers by employing X-ray photoelectron spectroscopy (XPS), Raman and photoluminescence (PL) spectroscopy, and atomic force microscopy (AFM). Although the large-area exfoliation of monolayer MoS_2_ proceeds on Ti at room temperature, a spontaneous reaction between the two materials alters most of the first layer of MoS_2_ to metallic molybdenum. We observe that the decomposition propagates even to the second MoS_2_ layer, but it is impeded by the presence of a third layer. Our results are supported by density functional theory (DFT) calculations, which demonstrate extremely strong titanium-induced hybridization of the MoS_2_ orbitals.

## Experimental Section

### UHV Exfoliation of MoS_2_ on Ti

The exfoliation of MoS_2_ on the Ti substrate was carried out inside a UHV chamber with a base pressure of 1 × 10^–10^ mbar. A schematic representation of the exfoliation process is shown in Figure 1. A detailed description of the UHV system can be found in our previous work.^30^ The bulk MoS_2_ crystal (Manchester Nanomaterials Ltd.) used for exfoliation was cut into 1 × 1 cm^2^ squares and pressed between two Si/SiO_2_ wafers to flatten it out. It was then attached to a SiO_2_/Si substrate using a UHV-compatible epoxy (Torr Seal), and the whole MoS_2_/SiO_2_/Si stack was mounted to a custom-made, stainless-steel sample holder and fixed by two spot-welded tantalum wires. The SiO_2_ (300 nm)/Si wafers used for exfoliation were cut into a desirable size and shape and cleaned with deionized water, acetone, and 2-propanol before being placed in a substrate holder. A fresh MoS_2_ surface was exposed by cleaving the bulk crystal under UHV using vacuum-compatible, double-sided Kapton adhesive tape. To improve the uniformity of the contact between the cleaved crystal and metallic substrate, we used ten layers of Kapton tape placed one on the top of another on a custom-made rocking sample holder (Figure S1), which can swing freely around the x- and *y*-axes. This setup helps the plane of the cleaved crystal align more uniformly with that of the substrate and reduces carbon contamination common to other elastic polymer stamps.

**Figure 1:**
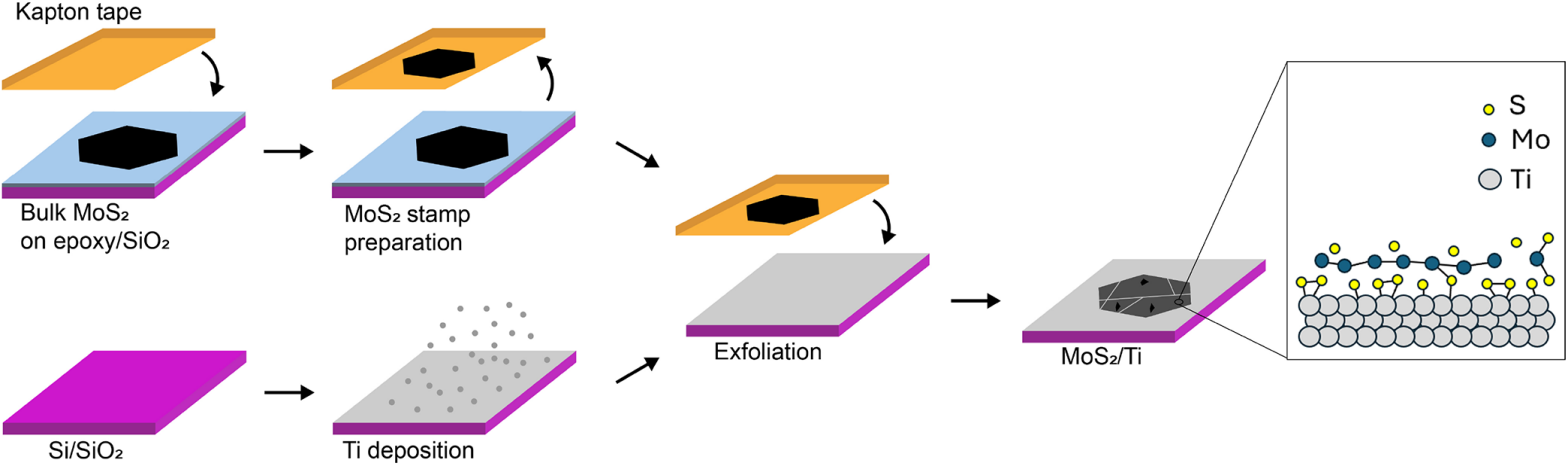
A schematic representation of the exfoliation process.

The UHV exfoliation was carried out using several manipulators and wobble sticks. The crystal cleavage and preparation of the metal substrate were performed in two separate parts of the same UHV chamber. The method proceeded as follows: (i) Kapton tape touched the bulk MoS_2_ crystal for cleaving; (ii) while the Kapton and the MoS_2_ crystal were in contact, e-beam evaporation of Ti onto the substrates commenced; (iii) near the end of the evaporation, the Kapton tape was retracted from the crystal, thus cleaving a fresh MoS_2_ surface; (iv) after evaporation of 7 nm of Ti, the cleaved MoS_2_ was brought in contact with the Ti-coated substrate in the minimum time required to move/transfer and align them inside the chamber.

### Optical Spectroscopy and Microscopy

Low-magnification optical images of MoS_2_ exfoliated on Ti were acquired in situ using a 5× objective mounted on a camera lens attached to the top viewport of the UHV system and stitched together to produce a macroscopic image of the exfoliated layers. High-magnification images were obtained with 50× and 100× objectives on a WITec alpha300 R confocal micro-Raman spectrometer operating under ambient conditions. This instrument was also used to measure Raman and PL spectra with 532 and 633 nm excitation, focused through a 100× Zeiss EC Epiplan-Neofluar objective and dispersed by an 1800 l/mm grating. Additional Raman spectra were collected with a LabRAM HR spectrometer (Horiba Jobin-Yvon) using 488, 514, 568, and 633 nm excitation, a 100× Olympus MPlan N objective, and a 600 l/mm grating. For all excitation wavelengths, the laser power was set to 0.5 mW.

ULF Raman measurements were performed with a home-built setup operating at 532 nm in a backscattering configuration. The system employed three BraggGate notch filters, enabling access to 7 cm^–1^. A 100× objective with N.A. = 0.9 was used for all measurements, producing a spot size of ≈0.5 × 0.5 μm^2^. To avoid heating, the laser power at the sample was kept below 1.5 mW. Spectra were collected with an Acton SpectraPro SP-2300 spectrometer (Princeton Instruments) equipped with an iDus 416A CCD (Andor) and an 1800 l/mm grating.

### X-ray Photoelectron Spectroscopy

The chemical composition of the exfoliated samples before and after exposure to the ambient environment or a controlled oxygen atmosphere was analyzed by XPS, using a Specs Phoibos 150 spectrometer equipped with a monochromatized Al Kα (*hν* = 1486.7 eV) source with a spot size of ≈1 mm^2^ and a base pressure of less than 10^–10^ mbar. A portable vacuum suitcase (base pressure ∼ 10^–10^ mbar) was used to transport the sample from the exfoliation chamber to the XPS machine for inspection of the composition before exposure to air. Note that during the transfer from the exfoliation chamber to the XPS system, the base pressure of the vacuum suitcase may increase slightly because the vacuum is maintained only by a non-evaporable getter pump. Micro-XPS measurements were performed using a NanoESCA instrument (Omicron) with spatial resolution ≈ 50 μm using monochromatized Al Kα (*hν* = 1486.7 eV) radiation. The instrument is equipped with photoemission electron microscopy that enables the localization of individual layers. The XPS attenuation factor used in the layer composition calculations was derived from experimentally measured dependence of the attenuation of the Au 4f XPS line intensity on the number of MoS_2_ adlayers. This experimental value is lower but more reliable than the theoretical attenuation factor calculated from the electron attenuation length based on several assumptions (density of the MoS_2_ monolayer, layer thickness, etc.).

### Atomic Force Microscopy and Kelvin Probe Force Microscopy

AFM images were acquired with an OmegaScope-R SPM/Optical system (HORIBA Scientific) operated in air in AC mode using ACCESS-FM-GG tips (Applied NanoStructures, Inc.). Kelvin probe force microscopy (KPFM) images were collected simultaneously in dual-pass FM-KPFM mode. The tip work function was calibrated against a freshly cleaved HOPG surface (work function = 4.6 eV^34^).

### DFT Calculations

We used the Quantum Espresso density functional theory code^35,36^ to compute the optimal structure, interaction energy, and projected density of states (PDOS) of 1L MoS_2_ deposited on the close-packed (0001) surface of hcp Ti. The slab thickness was set to 8 layers and the vacuum regime to a width of 12 Å, which was found sufficient to converge the interaction energy to a meV/Å^2^ precision. Full surface relaxation is performed on the freestanding Ti slab starting from a structure determined by the DFT lattice constant in the bulk crystal. During the structural optimization of the superstructure, the bottom half of the layers is kept fixed. We used the PBEsol density functional and a plane-wave cutoff energy of 680.3 eV (50 Ry). The k-point density for the 1L MoS_2_ was set to 12 × 12 × 1, and this was proportionally reduced in the superstructure corresponding to the supercell size ( $\sqrt{3}\times \sqrt{3}\;\mathrm{Mo}S_{2}$ on 2 × 2 in-plane Ti supercell, with compensating strain applied to the Ti slab to achieve commensurability). Following our previous work on other metals,^26^ we used the SSSP Precision library^37^ pseudopotentials for the Ti slab, while for the MoS_2_ slab, we chose the pslibrary 1.0.0 pseudopotentials.^38^

## Results and Discussion

MoS_2_ was exfoliated on Ti in a custom-made UHV system, in which *a* ≈ 7-nm-thick film of Ti was deposited on a 300 nm SiO_2_/Si substrate by e-beam evaporation. The optical image in Figure 2(a), taken after the exfoliation using an optical microscope attached to a UHV viewport, indicates a macroscopic-sized MoS_2_ layer of a positive optical contrast (black dashed trace) with visible discontinuities, cracks, and holes, which originate from the imperfections in the bulk MoS_2_ crystal, as previously observed on Au and Ag.^30^ A few small islands with thicker layers are also observed, as seen in Figure 2(b). The monolayer covers 76.3% of the area marked with the black dashed line, multilayers cover 0.8%, and the uncovered Ti accounts for 22.9% (Figure S2).

**Figure 2:**
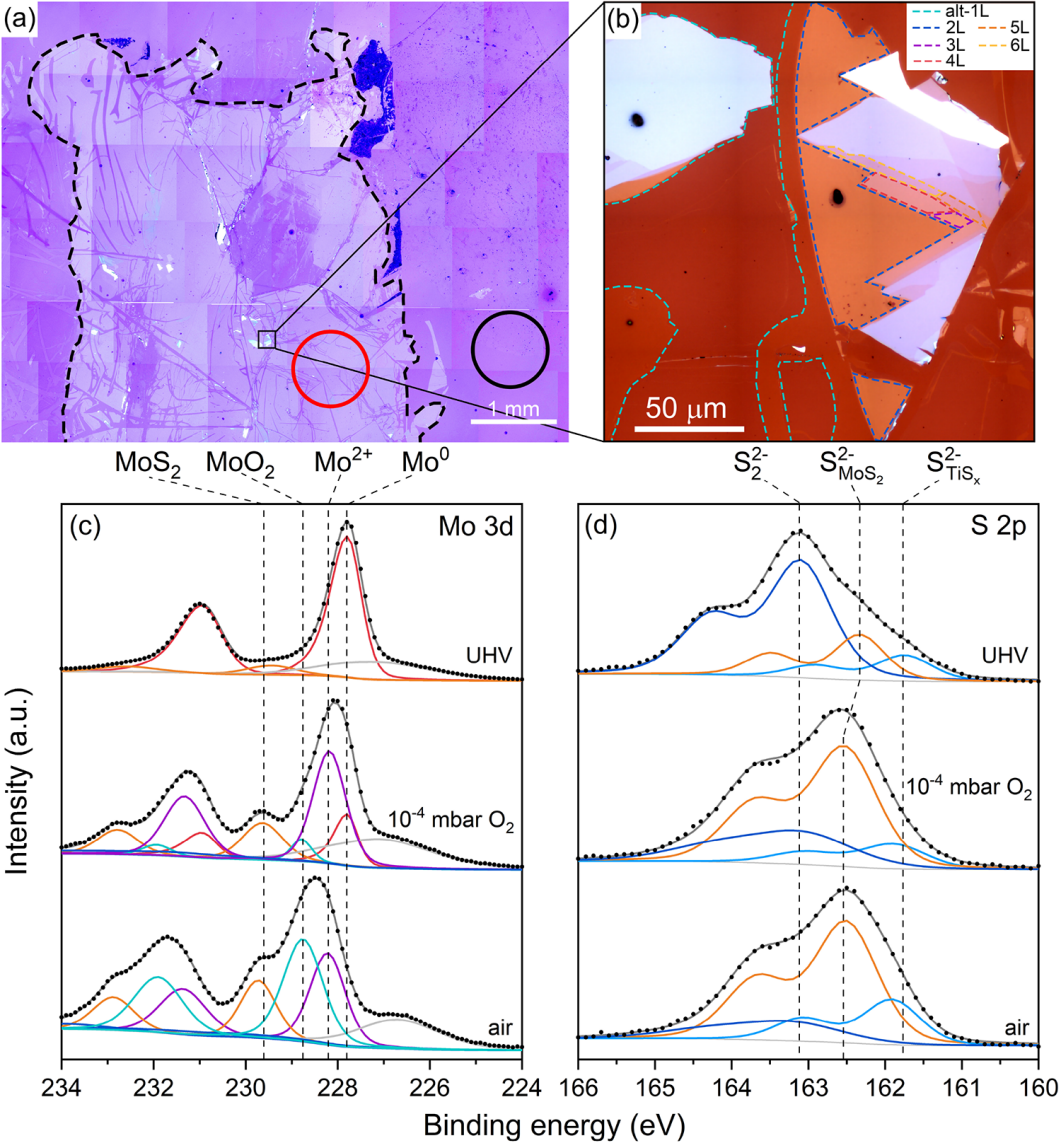
(a) Stitched optical in situ image of large-area MoS_2_ exfoliated on Ti under UHV (5× objective). The black dashed trace highlights the exfoliated area, and the red and black circles mark the areas investigated by large-spot XPS. (b) A high-magnification image of multilayer MoS_2_ on Ti taken in the ambient environment using a 100× objective. (c, d) XPS spectra of Mo 3d (c) and S 2p (d) core levels of the MoS_2_–Ti heterostructure acquired from the red circle area in (a).

To counteract the high reactivity of titanium, the sample was transferred to the XPS chamber without breaking the UHV to inspect the intrinsic interaction between MoS_2_ and Ti. The XPS survey (Figure S3) confirmed the presence of Mo and S in the region with the positive contrast and their absence outside of it (red and black circles in Figure 2(a), respectively). However, the XPS Mo 3d spectral region in Figure 2(c) obtained from the red circle region in Figure 2(a) is dominated by a doublet of Mo 3d with the Mo 3d_5/2_ binding energy at 227.8 eV, which corresponds to the Mo^0^ chemical state.^39^ Another Mo 3d_5/2_ line at 229.5 eV is assigned to the Mo^4+^ chemical state of MoS_2_, accounting only for ∼10% of the total Mo signal. As most of the molybdenum becomes metallic and the monolayer loses its MoS_2_ character, we will henceforth refer to it as the altered monolayer (alt-1L).

The S 2p XPS band in Figure 2(d) is relatively broad, and its shape differs from that of bulk or 1L MoS_2_ on gold (Figure S4). The S 2p XPS region was fitted by three doublets with the S 2p_3/2_ energies at 161.8, 162.5, and 163.1 eV. Lines at 161.8 and 163.1 eV are assigned to the sulfide (S_TiS__*x*_^2–^) atom and single-bonded disulfide (S_2_^2–^) group of TiS_3_.^40,41^ However, the disulfide/sulfide peak intensity ratio of ≈5 is significantly above the ideal value of 2 for TiS_3_, which indicates that in reality the structure is nonstoichiometric (TiS_*x*_) with an excess of disulfides. The remaining line of S 2p region at 162.5 eV is assigned to sulfur species (S_MoS__2_^2–^) in MoS_2_.^30,42,43^ The interfacial chemistry is constrained by the limited sulfur supply, the involvement of Mo, and the room temperature of the reaction, which makes the formation of a stoichiometric Ti–S phase unlikely. Since the TiS_*x*_ species originate from the interfacial reactions between Ti and MoS_2_, they are primarily limited by mass transport processes, which can modify their reaction kinetics compared to those in bulk systems.

Although the 163.1 eV band has previously also been attributed to Mo_*x*_S_*y*_ (0 < *y*/*x* < 2),^44^ a corresponding doublet belonging to this chemical species in the Mo 3d region between the Mo^0^ and Mo^4+^ chemical states is not resolved. The chemical states identified in the Mo 3d and S 2p spectra indicate that the exfoliated MoS_2_ exists as MoS_2_ or it decomposes into metallic Mo, both of which are well separated on the surface without intermediate states.

XPS performed at different takeoff angles revealed a decrease in relative intensity of the 163.1 eV component at low angles and no significant changes in the Mo 3d region (Figure S5). As the XPS signal from subsurface atoms is attenuated at low takeoff angles, angle-resolved measurements suggest that the TiS_*x*_ is preferentially located under the Mo species layer. The identification of TiS_*x*_ species from the Ti 2p XPS region is challenging, as the TiS_*x*_ contribution overlaps with that of the metallic Ti 2p line. In addition, the metallic Ti signal exhibits an asymmetric line shape and dominates the spectrum due to the relatively large thickness of the Ti substrate layer (Figure S6).

Chemical modification under UHV has been reported for Ti evaporated on MoS_2_ ^44–46^ and also for other common contact metals such as Cr, Sc, and Al, by a direct deposition of metals on bulk MoS_2_.^47,48^ Our results show that the interfacial reaction occurs even under the milder conditions of a simple mechanical contact between the two materials at room temperature. This approach ensures that the MoS_2_ modification occurs spontaneously because of the chemical reactivity of the two materials, rather than the damage incurred by the hot metal atoms upon impact. Indeed, the formation of the Ti–S bond is more thermodynamically favorable than the Mo–S bond, with the Gibbs free energy difference of −24.1 kcal/mol.^44,45,47,49^ Furthermore, we used DFT to calculate the interaction energy between MoS_2_ and Ti to be 1.05 eV per MoS_2_ unit cell, which is nearly seven times larger than that for MoS_2_ on Au (0.15 eV^26^). The calculated equilibrium distances between the bottom S and the first metal layer are 2.0 and 2.8 Å for Ti and Au, respectively. These insights from the theory, therefore, suggest that at least a partially covalent interaction between MoS_2_ and Ti is possible.

Titanium readily reacts with oxygen at room temperature, and atomically clean molybdenum surfaces similarly form adsorbed oxygen structures, while stoichiometric molybdenum oxides form only at elevated temperatures.^50–53^ We therefore examined oxidation in samples exposed to ambient air and low-pressure oxygen. Upon air exposure, metallic molybdenum in alt-1L MoS_2_ undergoes a chemical change, evidenced by the disappearance of the Mo^0^ component and the appearance of peaks at 228.2 and 228.7 eV, corresponding to Mo^2+^ and MoO_2_, respectively (Figure 2(c)).^39^ The Mo^2+^ signal may originate from MoO or MoS, consistent with previous reports on reduced/sulfurized molybdenum oxides and ion-bombarded MoS_2_.^54,55^ Oxidation also reduces the S 2p signal of TiS_*x*_ disulfide groups (Figure 2(d)), but less so under low-pressure oxygen. Air exposure increases the relative MoS_2_ intensity to ∼20% (from ∼10% in UHV), suggesting that oxygen-induced TiS_*x*_ decomposition releases sulfur, some of which reacts with metallic Mo to partially regenerate MoS_2_.

Sample exposure to oxygen at 10^–4^ mbar pressure yields a similar outcome to that for the sample exposed to the ambient conditions; however, some residual Mo^0^ component is still visible in the XPS of the former (Figure 2(c)). Disappearance of the disulfides after its interaction with oxygen is additional evidence for the nonstoichiometric TiS_*x*_ character because stoichiometric TiS_3_ is stable at room temperature in ambient conditions. This behavior indicates the presence of less stable disulfides that can be easily removed by oxygen. An analogy to our system with a thin sulfur layer on the Ti surface is the sulfur segregated on the clean titanium surface. The segregation of sulfur on the titanium surface proceeds by grain boundary diffusion to the surface, and then by surface diffusion to spread over the interface.^56^ The removal of segregated sulfur can be done by the exposure to low pressure oxygen at 20 °C.^57^ It should be noted that our samples were not heated, and therefore, the sulfur diffusion in Ti was limited. Sulfur diffusion into Ti bulk can play a role at higher temperatures above 400 °C.^56^

Next, we investigated the MoS_2_ multilayers on Ti following their exposure to air. To address their small lateral sizes, we used a micro-XPS instrument capable of confining the measurements to a specific location of the sample with a distinct number of MoS_2_ layers. The fitted spectra of the Mo 3d and S 2p core levels of alt-1L in Figure 3 are consistent with those measured by large-area XPS on the same sample. Isolated alt-1L signal contains oxidized Mo, S_2_^2–^, and S_TiS__*x*_^2–^ species arising from TiS_*x*_,^40,41,58^ and sulfur species (S_MoS__2_^2–^) from MoS_2_ ^30^ (Table S1). The main difference is the smaller fraction of MoS_2_ (∼6 ± 1%) with respect to the overall Mo signal for the micro-XPS measurement. This could be explained by inadvertent probing of regions of other MoS_2_ thicknesses, an issue exacerbated for the large-area XPS in Figure 2(c,d). The 2L and 3L spectra contain an increasingly higher fraction of MoS_2_ (Figure 3).

**Figure 3:**
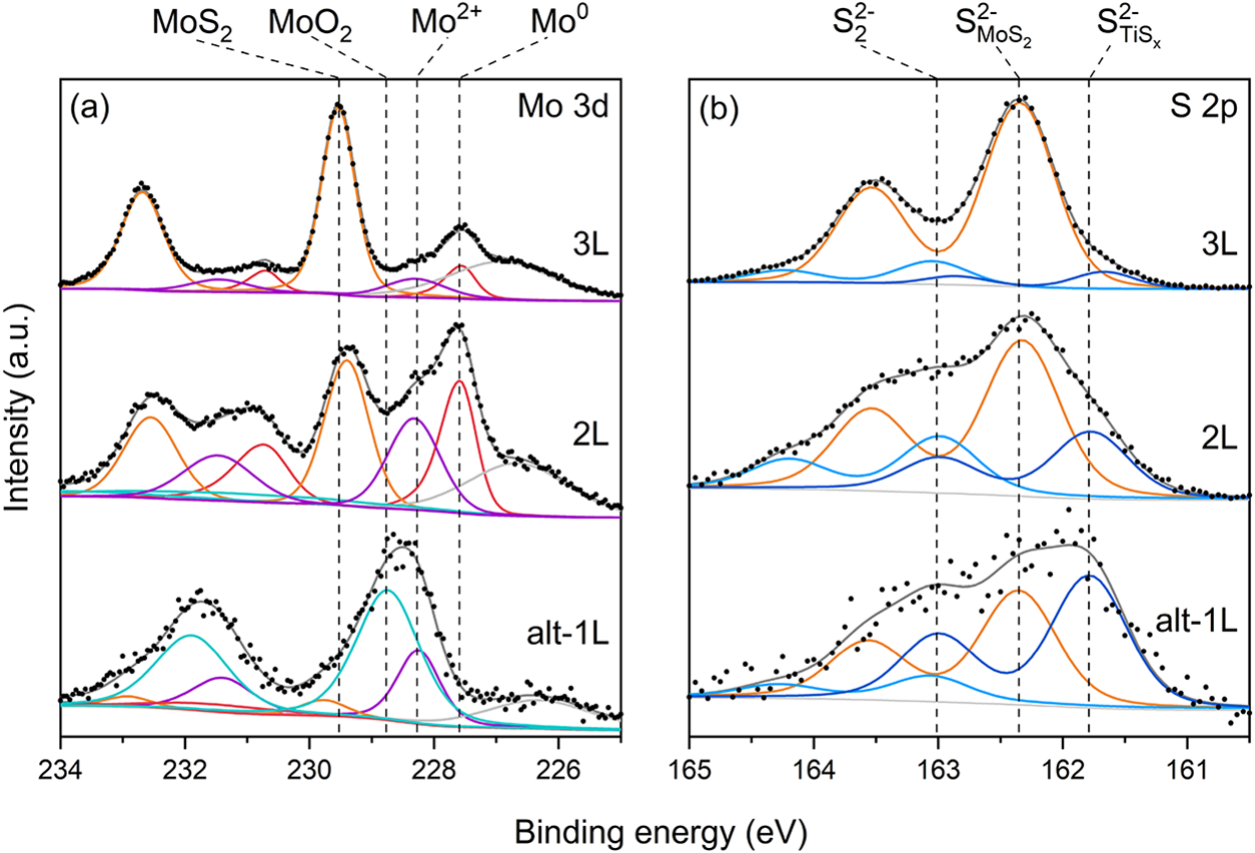
XPS spectra of (a) Mo 3d and (b) S 2p region of exfoliated MoS_2_ on Ti exposed to air taken from alt-1L, 2L, and 3L MoS_2_.

The MoS_2_ fraction for the 2L region, calculated from the Mo 3d area, was ≈36%. Assuming that the underlying alt-1L is in direct contact with Ti and contains the same amount of MoS_2_ as the air-exposed alt-1L (∼6%), and considering the experimentally determined attenuation factor of photoelectrons for 1L MoS_2_ of 0.6, we calculate that the second layer in 2L contains 54% of MoS_2_. In other words, approximately half of the second layer of the 2L region is modified by the interaction with titanium. As both Mo^0^ and Mo^2+^ species are observed, we infer that Mo^2+^ is primarily located beneath defective regions of the 2L, where oxygen can penetrate, whereas Mo^0^ is situated beneath the intact areas of MoS_2_. A similar interpretation applies to Ti, where metallic Ti and TiS_*x*_ species are expected to reside under intact MoS_2_, while oxidized TiO_*x*_ species are more likely associated with defective regions of the 2L.

The 3L region contains 76% MoS_2_ in total. Based on the data provided below, and assuming that the third (top) layer remains largely unaffected and the first layer retains ∼6% MoS_2_, we calculate that the second layer in 3L contains 78% MoS_2_, a higher fraction than in the second layer of the 2L region. This suggests that additional layers above the Ti-MoS_2_ interface reduce the extent of the interfacial reaction, implying that the assumption of a constant MoS_2_ fraction in the first layer of the 2L and 3L regions is likely inaccurate. A more plausible scenario is that decomposition of the first and second layers becomes less pronounced with increasing MoS_2_ thickness (e.g., 15 and 49% MoS_2_ in 2L, compared with 30 and 64% in 3L for the first and second layers, respectively; see Table S2). Crucially, the significant presence of metallic Mo in the 2L and 3L regions indicates that the bottommost layers undergo a spontaneous reaction with Ti, while the multilayer MoS_2_ protects the underlying metalized layers from oxidation (see the decrease in oxygen content in Table S1 and the Ti 2p and Figure S7). The Mo^2+^ peak likely arises from defective layers in which Mo atoms adopt a lower oxidation state.^55^

The presence of disorder in alt-1L and 2L is further corroborated by the featureless angle-resolved photoemission spectroscopy (ARPES) intensities in the (*k*_*x*_, *k*_*y*_)-space cuts. In contrast, 3L exhibits the characteristic MoS_2_ band structure, as shown in Figure S8 at a binding energy of *E*_B_ = 2.0 eV, providing evidence that it remains unaffected by the interaction with Ti.

To gain further insights into the structure of alt-1L MoS_2_, we calculated the Mo^0^ layer thickness from XPS spectra intensities using the Multiquant software and assuming a layer-by-layer model.^59^ The calculated thickness varied from 0.15 to 0.34 nm when measured at different positions of ≈1 mm X-ray spot size due to sample heterogeneity, confirming that most of the exfoliated material exists as a monolayer (alt-1L).

The decomposition of MoS_2_ is rooted in the strong interaction between MoS_2_ and Ti, as evidenced by the strong hybridization of the electronic structure calculated in Figures 4 and 5. The projected density of states of 1L MoS_2_, shown in Figure 4, reveals a significant difference between the freestanding 1L MoS_2_ and the 1L MoS_2_/Ti heterostructures. The band gap visible for freestanding 1L MoS_2_ completely disappears in the 1L MoS_2_/Ti heterostructure due to the emergence of new states that modify the electronic properties.

**Figure 4:**
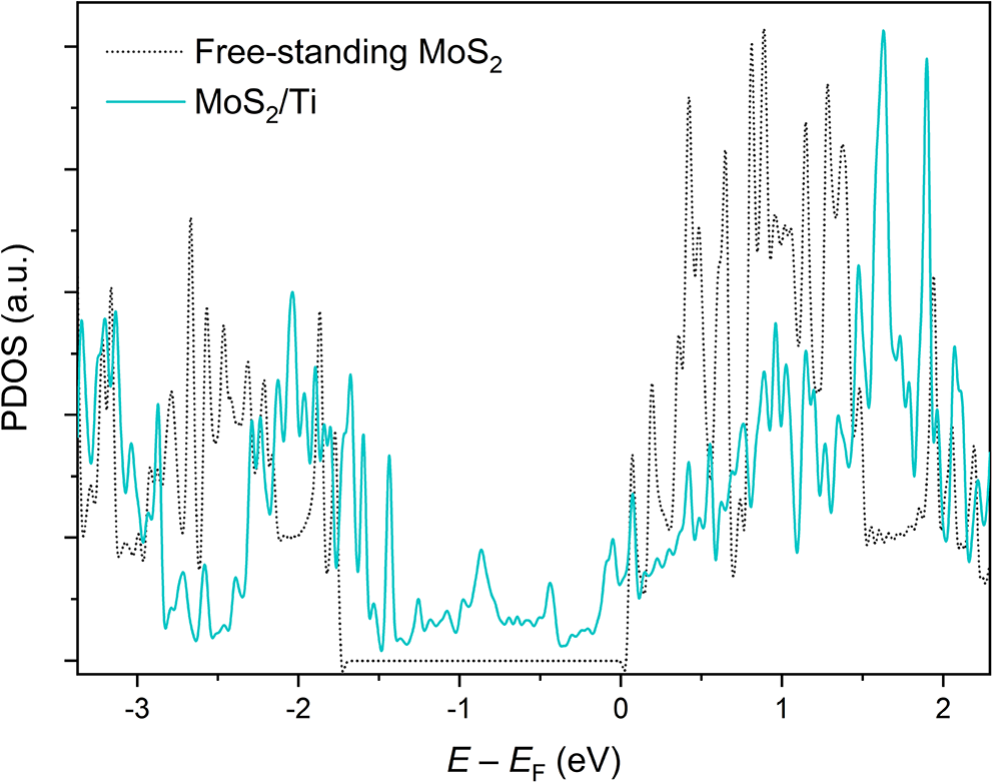
PDOS of freestanding MoS_2_ (black) and MoS_2_/Ti heterostructures (turquoise) calculated by DFT.

**Figure 5:**
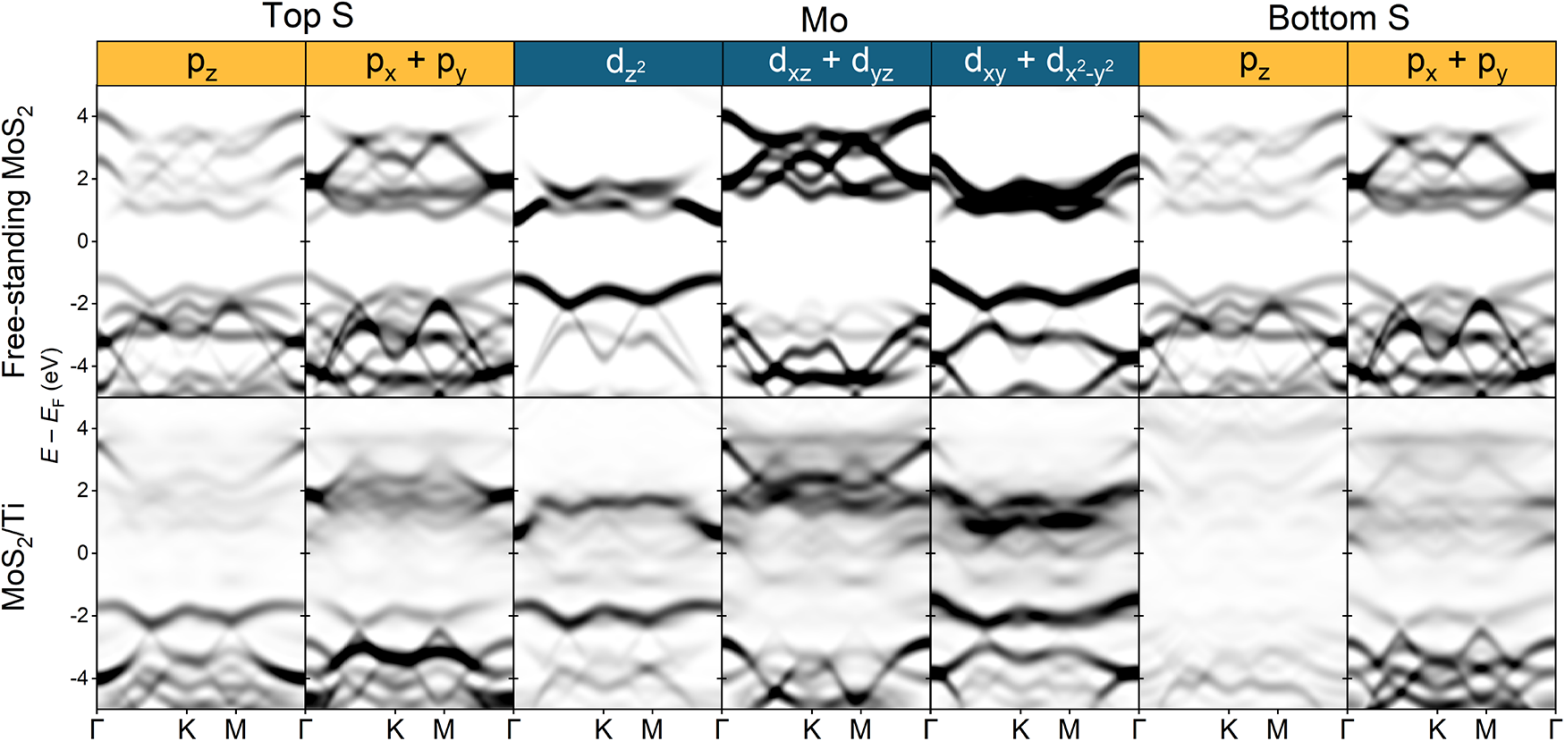
Orbital weights of the out-of-plane and in-plane S and Mo orbitals in the backfolded Brillouin zone for different atomic layers for freestanding MoS_2_ and MoS_2_/Ti heterostructures calculated by DFT.

The orbital weights of the out-of-plane and in-plane orbitals of Mo (d_*z* ^2^_, d_*xz*_+d_*yz*_, d_*xy*_+d_*x* ^2^–*y* ^2^_) and the top and bottom layer of S (p_*z*_, p_*x*+*y*_) shown in Figure 5 reveal pronounced shifting and blurring of the bands in the MoS_2_/Ti heterostructure when compared to the freestanding MoS_2_. The blurring of the bands occurs due to hybridization^26^ and has been previously observed in ARPES measurements of MoS_2_ on Au.^60^ There is a significant difference between the top and bottom S p_*z*_ orbitals, which has been suggested to disturb the van der Waals forces between the MoS_2_ layers, enabling large-area exfoliation.^26^ The in-plane orbitals are also significantly altered due to hybridization, which could promote the decomposition of MoS_2_, as they are responsible for the covalent bonds between Mo and S atoms.^61^

The layer-dependent decomposition of MoS_2_ interfaced with Ti prompted us to investigate the spectroscopic, electronic, and topographical properties of the samples by using Raman/PL spectroscopy, AFM, and KPFM. The alt-1L regions did not produce any Raman signal corresponding to MoS_2_ (Figure 6) because the monolayer decomposes into metallic Mo^0^ and TiS_*x*_ species upon mechanical contact in UHV and subsequently oxidizes to MoO_*x*_ upon air exposure, as confirmed by XPS, with only 6% of the original MoS_2_ remaining intact. The decomposition of alt-1L MoS_2_ is also revealed by ultralow-frequency Raman spectroscopy (Figure 6(a)), evidenced by the shift and/or disappearance of the first-order shear (S1) and breathing (B1) modes in MoS_2_/Ti compared with MoS_2_/SiO_2_.^28^ The absence of S1 and B1 peaks in 2L and their weakening in 3L confirms that the first MoS_2_ layer adjacent to Ti is almost completely decomposed, and the second layer is significantly damaged, consistent with the XPS results above. More generally, the Raman spectrum of an *N*-layer (*N* ≤ 6) flake on Ti resembles that of an (*N*–2)-layer flake on SiO_2_, as the bottom two layers are decoupled due to decomposition. These observations are consistent with trends reported on Au.^28^

**Figure 6:**
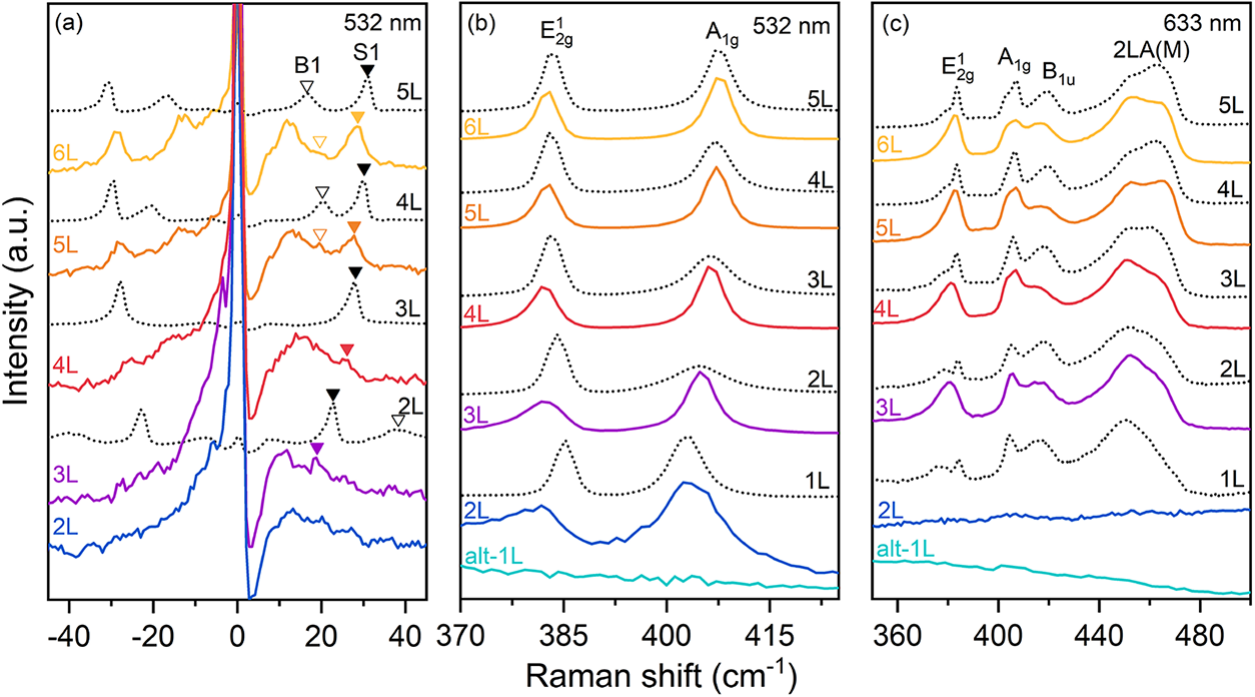
Layer-dependent Raman spectra of MoS_2_ on Ti (solid color curves), compared with MoS_2_ on SiO_2_ (dotted black curves). (a) Ultralow-frequency Raman spectra and (b) high-frequency Raman spectra taken at the 532 nm excitation wavelength. (c) High-frequency Raman spectra taken at the 633 nm excitation wavelength.

The high-frequency Raman spectrum of 2L MoS_2_ on Ti (blue in Figure 6(b)), measured with a 532 nm excitation wavelength, contains both the main E_2*g*_^1^ and A_1g_ MoS_2_ modes. However, it is more similar to 1L (rather than 2L) on SiO_2_, since the underlying alt-1L layer can no longer be considered to be MoS_2_. Both Raman modes are broadened, which we attribute to a high density of structural defects as only ≈ 54% of the layer is preserved as MoS_2_ according to the XPS.^62^ The presence of the defects is further evidenced by the appearance of a broad LA­(M) mode around 220 cm^–1^ (Figure S9), typical for defective monolayer MoS_2_.^62^ The E_2*g*_^1^ mode of 2L MoS_2_ on Ti is downshifted, likely due to defects^63^ and tensile strain induced by the underlying alt-1L, metallic Mo^0^, Ti, and TiS_*x*_. As the thickness increases, the peak positions converge to those of MoS_2_ on SiO_2_ with (*N*–1) layers, demonstrating that the top layers remain largely unaffected by the Ti interaction.

When MoS_2_ is excited with the 633 nm laser, several additional peaks appear in the Raman spectrum due to resonant conditions.^64,65^ For thicker MoS_2_ on Ti, the Raman signal reappears and is consistent with the literature for (*N*–1) layers of MoS_2_ on SiO_2_.^64,65^ However, Figure 6(c) shows that neither the alt-1L nor the 2L produces any Raman signal. While no Raman is expected from the decomposed monolayer (alt-1L), as discussed above, the strong interaction, strain, and disorder introduced to 2L may increase the band gap above the 1.96 eV (633 nm) excitation energy, thereby significantly reducing the cross-section of the Raman scattering.^66^ Nevertheless, we assume that underlying metallic Ti or Mo^0^, protected by the 54% intact MoS_2_, can play a decisive role in quenching the Raman signal (for 633 nm laser) by transferring charge or metallic screening, as it has been reported in the case of MoS_2_ directly exfoliated on Au by earlier works.^67,68^ The structural disintegration caused by Ti and/or the underlying metal residues can also influence the PL spectra. They likely quench the PL signals in alt-1L and 2L MoS_2_ (Figure S10). We also did not observe any PL signals up to 6L. We presume that the transition of band gap type from direct to indirect with increasing layer number has played a primary role, as these layers were either less (in 3L, 78% MoS_2_ remains intact) or not at all affected by the Ti substrate.^69^ We observed a similar trend to the results obtained using a 532 nm laser line for other excitation wavelengths (488, 514, and 568 nm), which are expected to be above the band gap of MoS_2_, Figure S11.

The AFM topography and KPFM measurements performed on the sample under ambient conditions are shown in Figure 7. The 2L MoS_2_ region is found to be higher than the adjacent 3L and 4L, likely due to the expected volume expansion of the underlying reacted Ti and the alt-1L layer. This is consistent with the XPS results, which confirm that 2L MoS_2_ only partially protects Ti from oxidation, while the thicker 3L and 4L MoS_2_ layers passivate the surface. A stark difference between the 2L, 3L, and 4L is also observed in the KPFM image, where the work function of the 2L is approximately 550 meV lower than that of the surrounding layers, indicating an upshift of the Fermi level in contact with the underlying titanium oxide as reported earlier.^70^

**Figure 7:**
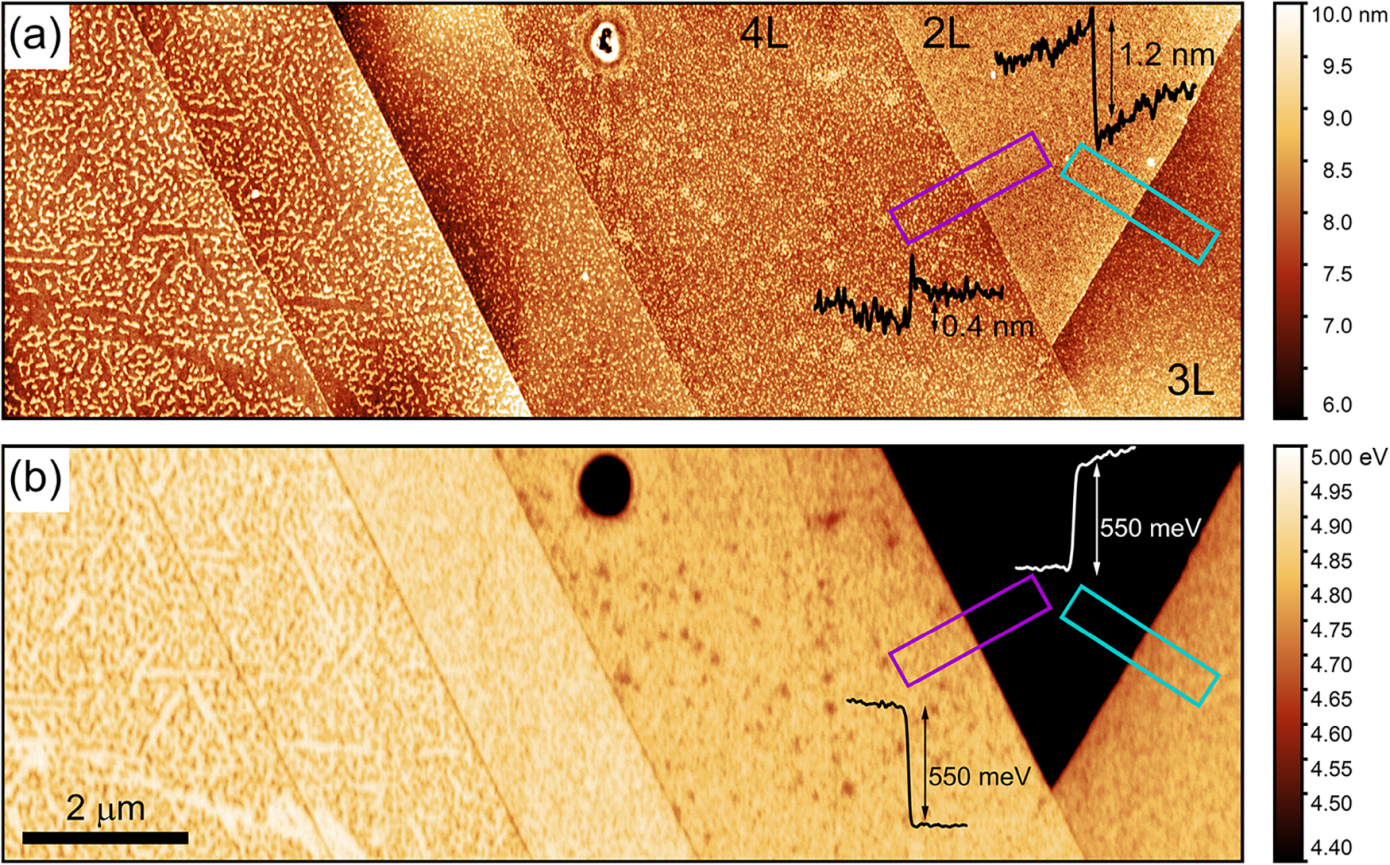
(a) Topography measured with AFM on a multilayer MoS_2_/Ti area with its corresponding work function map shown in (b). The line profiles of the height and work function marked in (a) and (b) are shown as insets.

## Conclusion

In conclusion, the exfoliation of MoS_2_ on Ti under UHV yields macroscopic layers comparable in size to those obtained on Au and other metals. Unlike noble metals, however, the strong chemical reactivity of Ti at room temperature leads to decomposition of the first MoS_2_ monolayer to metallic Mo and the formation of nonstoichiometric TiS_*x*_ at the interface upon simple physical contact, as evidenced by XPS, Raman spectroscopy, and supported by DFT calculations. Upon air exposure, metallic Mo and interfacial Ti oxidize, TiS_*x*_ disintegrates, and the partial recovery of MoS_2_ occurs. Notably, oxidation is suppressed in bilayer MoS_2_, while trilayers and thicker flakes provide effective protection of the interface. However, quantitative analysis reveals that even the second layer is partially affected by Ti, although the extent diminishes as additional MoS_2_ layers are present. These insights could enable new studies on how interfacial reaction products influence nanoscale device performance and could be extended to other metals commonly used for contact engineering in 2D materials. While the transfer of MoS_2_ monolayers has been reported previously, where their intrinsic properties are restored after transfer,^71^ our findings suggest that 2D materials could also be directly exfoliated onto patterned Ti substrates, offering a potential route for device integration. This is particularly applicable to reactive metals with high melting points, such as Cr, Ta, and Hf, where conventional high-energy deposition often damages 2D materials.

## Supplementary Material



## Data Availability

The data underlying this study are openly available from the HeyRACK repository at https://doi.org/10.48700/datst.7nrq7-mqe46.

## Notes

Sample holder image; elemental concentrations calculated from XPS; calculated MoS_2_ fractions; exfoliation yield expressed as a percentage of the total area; XPS survey spectra; XPS spectra of bulk MoS_2_ and MoS_2_ on Au(111); takeoff angle XPS measurements; Ti 2p XPS spectra; micro-ARPES for alt-1L, 2L, and 3L MoS_2_ on Ti; 2L MoS_2_ Raman spectrum; PL measurements; and layer-dependent Raman spectra (PDF)

## References

[1] Baugher B. W. H., Churchill H. O., Yang Y., Jarillo-Herrero P. (2013). Intrinsic
electronic transport properties of high-quality monolayer and bilayer
MoS_2_. Nano Lett..

[2] Mak K. F., Lee C., Hone J., Shan J., Heinz T. F. (2010). Atomically thin
MoS_2_: a new direct-gap semiconductor. Phys. Rev. Lett..

[3] Xiao D., Liu G.-B., Feng W., Xu X., Yao W. (2012). Coupled spin
and valley physics in monolayers of MoS_2_ and other group-VI
dichalcogenides. Phys. Rev. Lett..

[4] Zeng H., Dai J., Yao W., Xiao D., Cui X. (2012). Valley polarization
in MoS_2_ monolayers by optical pumping. Nat. Nanotechnol..

[5] Wu W., Wang L., Li Y., Zhang F., Lin L., Niu S., Chenet D., Zhang X., Hao Y., Heinz T. F., Hone J., Lin Wang Z. (2014). Piezoelectricity of single-atomic-layer
MoS_2_ for energy conversion and piezotronics. Nature.

[6] Chen W., Qiu Y., Babichuk I. S., Chang Y., Zhou R., He Z., Liu Y., Zhang J., Babichuk I. V., Tiutiunnyk A., Laroze D., V Brus V., Yang J. (2024). Improving the Strain
control performance of MoS_2_ monolayer to develop flexible
electronics. Adv. Eng. Mater..

[7] Lan H.-Y., Yang S.-H., Kantre K.-A., Cott D., Tripathi R., Appenzeller J., Chen Z. (2025). Reliability of high-performance
monolayer
MoS_2_ transistors on scaled high-*κ* HfO_2_. npj 2D Mater. Appl..

[8] Zhou Z., Lv J., Tan C., Yang L., Wang Z. (2024). Emerging frontiers
of 2D transition metal dichalcogenides in photovoltaics solar cell. Adv. Funct. Mater..

[9] Wang H., Hou S., Feng W., Li D., Liu J., Yang W., Huang S., Li F., Zhao X., Chen F., Huang C., Pan Y. (2025). An aptamer-based
MoS_2_ field-effect
transistor biosensor with high sensitivity for cytokine detection. Mater. Today Nano.

[10] Somvanshi D., Kallatt S., Venkatesh C., Nair S., Gupta G., Anthony J. K., Karmakar D., Majumdar K. (2017). Nature of carrier injection
in metal/2D-semiconductor interface and its implications for the limits
of contact resistance. Phys. Rev. B.

[11] Kim C., Moon I., Lee D., Choi M. S., Ahmed F., Nam S., Cho Y., Shin H.-J., Park S., Yoo W. J. (2017). Fermi level
pinning at electrical metal contacts of monolayer molybdenum dichalcogenides. ACS Nano.

[12] Yoon J., Park W., Bae G.-Y., Kim Y., Jang H. S., Hyun Y., Lim S. K., Kahng Y. H., Hong W.-K., Lee B. H., Ko H. C. (2013). Highly flexible and transparent multilayer
MoS_2_ transistors with graphene electrodes. Small.

[13] Schock R. T. K., Neuwald J., Möckel W., Kronseder M., Pirker L., Remškar M., Hüttel A. K. (2023). Non-Destructive
Low-Temperature Contacts to MoS_2_ Nanoribbon and Nanotube
Quantum Dots. Adv. Mater..

[14] Das S., Chen H.-Y., Penumatcha A. V., Appenzeller J. (2013). High performance
multilayer MoS_2_ transistors with scandium contacts. Nano Lett..

[15] Popov I., Seifert G., Tománek D. (2012). Designing electrical contacts to
MoS_2_ monolayers: a computational study. Phys. Rev. Lett..

[16] Kim C., Lee K. Y., Moon I., Issarapanacheewin S., Yoo W. J. (2019). Metallic contact induced van der
Waals gap in a MoS_2_ FET. Nanoscale.

[17] Kwon G., Kim H.-S., Jeong K., Oh S., Kim D., Koh W., Park H., Im S., Cho M.-H. (2025). Reconfiguring van
der Waals Metal-Semiconductor Contacts via Selenium Intercalation/Deintercalation
Post-Treatment. ACS Nano.

[18] Liu Y., Stradins P., Wei S.-H. (2016). Van der
Waals metal-semiconductor
junction: Weak Fermi level pinning enables effective tuning of Schottky
barrier. Sci. Adv..

[19] Magda G. Z., Petö J., Dobrik G., Hwang C., Biró L. P., Tapasztó L. (2015). Exfoliation of large-area transition metal chalcogenide
single layers. Sci. Rep..

[20] Desai S. B., Madhvapathy S. R., Amani M., Kiriya D., Hettick M., Tosun M., Zhou Y., Dubey M., Ager J. W., Chrzan D., Javey A. (2016). Gold-mediated exfoliation of ultralarge
optoelectronically-perfect monolayers. Adv.
Mater..

[21] Velický M., Donnelly G. E., Hendren W. R., McFarland S., Scullion D., DeBenedetti W. J., Correa G. C., Han Y., Wain A. J., Hines M. A., A Muller D., S Novoselov K., Abruña D., M Bowman R., J G Santos E., Huang F. (2018). Mechanism of gold-assisted exfoliation of centimeter-sized transition-metal
dichalcogenide monolayers. ACS Nano.

[22] Huang Y., Pan Y.-H., Yang R., Bao L.-H., Meng L., Luo H.-L., Cai Y.-Q., Liu G.-D., Zhao W.-J., Zhou Z., Wu L.-M., Zhu Z.-L., Huang M., Liu L.-W., Liu L., Cheng P., Wu K.-H., Tian S.-B., Gu C.-Z., Shi Y.-G., Guo Y.-F., Cheng Z. G., Hu J.-P., Zhao L., Yang G.-H., Sutter E., Sutter P., Wang Y.-L., Ji W., Zhou X.-J., Gao H.-J. (2020). Universal mechanical exfoliation
of large-area 2D crystals. Nat. Commun..

[23] Pollmann E., Sleziona S., Foller T., Hagemann U., Gorynski C., Petri O., Madauß L., Breuer L., Schleberger M. (2021). Large-area,
two-dimensional MoS_2_ exfoliated on gold: Direct experimental
access to the metal-semiconductor interface. ACS Omega.

[24] Heyl M., Grützmacher S., Rühl S., Ligorio G., Koch N., List-Kratochvil E. J. (2022). Low Temperature Heating of Silver-Mediated Exfoliation
of MoS_2_. Adv. Mater. Interfaces.

[25] Panasci S. E., Schilirò E., Migliore F., Cannas M., Gelardi F., Roccaforte F., Giannazzo F., Agnello S. (2021). Substrate impact on
the thickness dependence of vibrational and optical properties of
large area MoS_2_ produced by gold-assisted exfoliation. Appl. Phys. Lett..

[26] Hanušová M., Pirker L., Vondráček M., Valeš V., Cheung C. K., Natera Cordero N., Carl A., Zólyomi V., Koltai J., Sotiriou I., Zscharschuch J., Erbe A., Gorbachev R., Honolka J., Frank O., Velický M. (2025). Hybridization Directionality Governs the Interaction
Strength between MoS_2_ and Metals. Nano Lett..

[27] Pirker L., Honolka J., Velický M., Frank O. (2024). When 2D Materials Meet
Metals. 2D Mater..

[28] Ziewer J., Ghosh A., Hanušová M., Pirker L., Frank O., Velickỳ M., Grüning M., Huang F. (2025). Strain-Induced Decoupling Drives
Gold-Assisted Exfoliation of Large-Area
Monolayer 2D Crystals. Adv. Mater..

[29] Velický M., Donnelly G. E., Hendren W. R., DeBenedetti W. J., Hines M. A., Novoselov K. S., Abruña H. D., Huang F., Frank O. (2020). The intricate love affairs between
MoS_2_ and metallic substrates. Adv.
Mater. Interfaces.

[30] Haider G., Gastaldo M., Karim B., Plsek J., Varade V., Volochanskyi O., Vejpravova J., Kalbac M. (2024). Highly efficient bulk-crystal-sized
exfoliation of 2D materials under ultrahigh vacuum. ACS Appl. Electron. Mater..

[31] Grubišić-Čabo A., Michiardi M., Sanders C. E., Bianchi M., Curcio D., Phuyal D., Berntsen M. H., Guo Q., Dendzik M. (2023). In Situ Exfoliation
Method of Large-Area 2D Materials. Adv. Sci..

[32] Sun Z., Han X., Cai Z., Yue S., Geng D., Rong D., Zhao L., Zhang Y.-Q., Cheng P., Chen L., Zhou X., Huang Y., Wu K., Feng B. (2022). Exfoliation
of 2D van der Waals crystals in ultrahigh vacuum for interface engineering. Sci. Bull..

[33] Liu G. (2025). Theoretical
Study of Ti and Cr as Candidate Assisted Metals for Mechanical Exfoliation
of Monolayer Transition Metal Dichalcogenides. Sci. Rep..

[34] Kim J. W., Kim A., Hwang H. U., Kim J. H., Choi S., Koch N., Shin D., Zhao Z., Liu F., Choi M., Lee K. M., Park Y. (2023). Work function measurement by ultraviolet
photoelectron spectroscopy: Versailles project on advanced materials
and standards interlaboratory study. J. Vac.
Sci. Technol. A.

[35] Giannozzi P., Baroni S., Bonini N., Calandra M., Car R., Cavazzoni C., Ceresoli D., Chiarotti G. L., Cococcioni M., Dabo I., Dal Corso A., de Gironcoli S., Fabris S., Fratesi G., Gebauer R., Gerstmann U., Gougoussis C., Kokalj A., Lazzeri M., Martin-Samos L., Marzari N., Mauri F., Mazzarello R., Paolini S., Pasquarello A., Paulatto L., Sbraccia C., Scandolo S., Sclauzero G., Seitsonen A. P., Smogunov A., Umari P., Wentzcovitch R. M. (2009). QUANTUM
ESPRESSO: A Modular and Open-source Software Project for Quantum Simulations
of Materials. J. Phys.: Condens. Matter.

[36] Giannozzi P., Andreussi O., Brumme T., Bunau O., Buongiorno
Nardelli M., Nardelli M. B., Calandra M., Car R., Cavazzoni C., Ceresoli D., Cococcioni M., Colonna N., Carnimeo I., Dal Corso A., Corso A. D., de Gironcoli S., Delugas P., DiStasio R. A., Jr R. A. D., Ferretti A., Floris A., Fratesi G., Fugallo G., Gebauer R., Gerstmann U., Giustino F., Gorni T., Jia J., Kawamura M., Ko H.-Y., Kokalj A., Küçükbenli E., Lazzeri M., Marsili M., Marzari N., Mauri F., Nguyen N. L., Nguyen H.-V., Otero-de-la-Roza A., Paulatto L., Paulatto L., Poncé S., Poncé S., Rocca D., Rocca D., Sabatini R., Sabatini R., Santra B., Santra B., Schlipf M., Schlipf M., Seitsonen A. P., Seitsonen A. P., Smogunov A., Smogunov A., Timrov I., Timrov I., Thonhauser T., Thonhauser T., Umari P., Umari P., Vast N., Vast N., Wu X., Wu X., Baroni S. (2017). Advanced Capabilities for Materials Modelling with
QUANTUM ESPRESSO. J. Phys.: Condens. Matter.

[37] Prandini G., Marrazzo A., Castelli I. E., Mounet N., Marzari N. (2018). Precision
and Efficiency in Solid-State Pseudopotential Calculations.. npj Comput. Mater..

[38] Dal
Corso A. (2014). Pseudopotentials Periodic Table: From H to Pu. Comput. Mater. Sci..

[39] Werfel F., Minni E. (1983). Photoemission study
of the electronic structure of Mo and Mo oxides. J. Phys. C: Solid State Phys..

[40] Hawkins C. G., Whittaker-Brooks L. (2018). Controlling sulfur vacancies in TiS_2–*x*
_ cathode insertion hosts via the conversion of TiS_3_ nanobelts for energy-storage applications. ACS Appl. Nano Mater..

[41] Fleet M., Harmer S., Liu X., Nesbitt H. (2005). Polarized X-ray absorption
spectroscopy and XPS of TiS_3_: S K-and Ti L-edge XANES and
S and Ti 2p XPS. Surf. Sci..

[42] Santoni A., Rondino F., Malerba C., Valentini M., Mittiga A. (2017). Electronic structure of Ar^+^ ion-sputtered
thin-film MoS_2_: A XPS and IPES study. Appl. Surf. Sci..

[43] Baker M., Gilmore R., Lenardi C., Gissler W. (1999). XPS investigation
of
preferential sputtering of S from MoS_2_ and determination
of MoS_
*x*
_ stoichiometry from Mo and S peak
positions. Appl. Surf. Sci..

[44] Freedy K. M., McDonnell S. J. (2020). Contacts
for molybdenum disulfide: interface chemistry
and thermal stability. Materials.

[45] McDonnell S., Smyth C., Hinkle C. L., Wallace R. M. (2016). MoS_2_–titanium
contact interface reaction. ACS Appl. Mater.
Interfaces.

[46] Schauble K., Zakhidov D., Yalon E., Deshmukh S., Grady R. W., Cooley K. A., McClellan C. J., Vaziri S., Passarello D., Mohney S. E., F Toney M., Sood A. K., Salleo A., Pop E. (2020). Uncovering the effects of metal contacts on monolayer MoS_2_. ACS Nano.

[47] McGovern I., Dietz E., Rotermund H., Bradshaw A., Braun W., Radlik W., McGilp J. (1985). Soft X-ray photoemission spectroscopy
of metal-molybdenum bisulphide interfaces. Surf.
Sci..

[48] Smyth C. M., Addou R., McDonnell S., Hinkle C. L., Wallace R. M. (2016). Contact
Metal-MoS_2_ Interfacial Reactions and Potential Implications
on MoS_2_-Based Device Performance. J. Phys. Chem. C.

[49] Smart R. S. C., Skinner W. M., Gerson A. R. (1999). XPS of sulphide
mineral surfaces:
metal-deficient, polysulphides, defects and elemental sulphur. Surf. Interface Anal..

[50] Bauer E., Poppa H. (1983). On the adsorption of
oxygen on the Mo{110} surface and its vicinals. Surf. Sci..

[51] Kennett H., Lee A. (1975). The initial oxidation of molybdenum I I. LEED and RHEED observations
on (110) molybdenum. Surf. Sci..

[52] Kennett H., Lee A. (1975). The initial oxidation
of molybdenum II. RHEED observations on (100)
and (111) surfaces. Surf. Sci..

[53] Zhang C., Van Hove M., Somorjai G. (1985). The interaction
of oxygen with the
Mo(100) and Mo(111) single-crystal surfaces: chemisorption and oxidation
at high temperatures. Surf. Sci. Lett..

[54] Spevack P. A., McIntyre N. (1993). A Raman and XPS investigation
of supported molybdenum
oxide thin films. 2. Reactions with hydrogen sulfide. J. Phys. Chem. A.

[55] McIntyre N., Spevack P., Beamson G., Briggs D. (1990). Effects of argon ion
bombardment on basal plane and polycrystalline MoS_2_. Surf. Sci..

[56] Outlaw R. A., Lee W. S., Hoekje S. J., Sankaran S. N. (1994). Sulfur
segregation
in titanium and selected titanium alloys. Appl.
Surf. Sci..

[57] Reibel R., Schneider S., Cruguel H., Lapeyre J., Smith R. J. (1999). A kinetic
model for the removal of sulfur from polycrystalline titanium surfaces
exposed to oxygen. Surf. Rev. Lett..

[58] Lindic M.-H., Pecquenard B., Vinatier P., Levasseur A., Martinez H., Gonbeau D., Petit P.-E., Ouvrard G. (2005). Characterization
of rf sputtered TiO_
*y*
_S_
*z*
_ thin films. Thin Solid Films.

[59] Mohai M. (2004). XPS MultiQuant:
Multimodel XPS quantification software. Surf.
Interface Anal..

[60] Bruix A., Miwa J. A., Hauptmann N., Wegner D., Ulstrup S., Grønborg S. S., Sanders C. E., Dendzik M., Grubišić
Čabo A., Bianchi M., V Lauritsen J., A Khajetoorians A., Hammer B., Hofmann P. (2016). MoS_2_ on
Au(111): Band gap renormalization and substrate interaction. Phys. Rev. B.

[61] Cappelluti E., Roldán R., Silva-Guillén J., Ordejón P., Guinea F. (2013). Tight-binding model and direct-gap/indirect-gap
transition
in single-layer and multilayer MoS_2_. Phys. Rev. B.

[62] Fujisawa K., Carvalho B. R., Zhang T., Perea-Lopez N., Lin Z., Carozo V., Ramos S. L., Kahn E., Bolotsky A., Liu H., Laura Elías A., Terrones M. (2021). Quantification and
healing of defects in atomically thin molybdenum disulfide: beyond
the controlled creation of atomic defects. ACS
Nano.

[63] Mignuzzi S., Pollard A. J., Bonini N., Brennan B., Gilmore I. S., Pimenta M. A., Richards D., Roy D. (2015). Effect of disorder
on Raman scattering of single-layer MoS_2_. Phys. Rev. B.

[64] Chakraborty B., Matte H. R., Sood A., Rao C. (2013). Layer-dependent
resonant
Raman scattering of a few layer MoS_2_. J. Raman Spectrosc..

[65] Gołasa K., Grzeszczyk M., Bożek R., Leszczyński P., Wysmołek A., Potemski M., Babiński A. (2014). Resonant Raman
scattering in MoS_2_From bulk to monolayer. Solid State Commun..

[66] Yang T., Huang X., Zhou H., Wu G., Lai T. (2016). Excitation
mechanism of A_1g_ mode and origin of nonlinear temperature
dependence of Raman shift of CVD-grown mono-and few-layer MoS_2_ films. Opt. Express.

[67] Velický M., Rodriguez A., Bousa M., Krayev A. V., Vondracek M., Honolka J., Ahmadi M., Donnelly G. E., Huang F., Abruna H. D., S Novoselov K., Frank O. (2020). Strain and charge doping
fingerprints of the strong interaction between monolayer MoS_2_ and gold. J. Phys. Chem. Lett..

[68] Rodriguez A., Velickỳ M., Řáhová J., Zólyomi V., Koltai J., Kalbáč M., Frank O. (2022). Activation
of Raman modes in monolayer transition metal dichalcogenides
through strong interaction with gold. Phys.
Rev. B.

[69] Splendiani A., Sun L., Zhang Y., Li T., Kim J., Chim C.-Y., Galli G., Wang F. (2010). Emerging photoluminescence in monolayer
MoS_2_. Nano Lett..

[70] Lattyak C., Gehrke K., Vehse M. (2022). Layer-thickness-dependent
work function
of MoS_2_ on metal and metal oxide substrates. J. Phys. Chem. C.

[71] Sahu S., Haider G., Rodriguez A., Plšek J., Mergl M., Kalbáč M., Frank O., Velickỳ M. (2023). Large-Area Mechanically-Exfoliated
Two-Dimensional
Materials on Arbitrary Substrates. Adv. Mater.
Technol..

