# Supplementary material for: Spontaneous Room-Temperature Solid-State Reaction at the MoS2/Ti Interface: Implications for Contact Engineering
Source: ACS Appl Nano Mater. 2025 Dec 5;8(50):24022–32. doi: 10.1021/acsanm.5c04350 (PMC12723649; doi:10.1021/acsanm.5c04350)
Supplement: Supplementary file 1 [file an5c04350_si_001.pdf]

Supporting Information: Spontaneous Room-Temperature Solid-State Reaction at the MoS_2/Ti Interface: Implications for Contact Engineering

Bazlul Karim,[†,⊥] Luka Pirker,[†,⊥] Jan Plšek,[†] Václav Valeš,[‡] Martin Vondráček,[‡] Michaela Hanušová,[†,¶] Viktor Zólyomi,[§] Michele Gastaldo,[†] Abhilash Bajpai,[†] Jakob Ziewer,[‖] Fumin Huang,[‖] Jan Honolka,[‡] Otakar Frank,[†] Matěj Velický,[*,†] and Martin Kalbáč[*,†]

†*J. Heyrovský Institute of Physical Chemistry, Czech Academy of Sciences, Dolejškova 2155/3, 182 23 Prague 8, Czech Republic*

‡*Institute of Physics, Czech Academy of Sciences, Na Slovance 1999/2, 182 21 Prague 8, Czech Republic*

¶*Faculty of Chemical Engineering, University of Chemistry and Technology, Prague, Technická 5, 166 28 Prague 6, Czech Republic*

§*Hartree Centre, STFC Daresbury Laboratory, Daresbury WA4 4AD, United Kingdom*

‖*Centre for Quantum Materials and Technologies, School of Mathematics and Physics, Queen's University Belfast, University Road, Belfast, BT7 1NN, United Kingdom*

⊥*Contributed equally to this work*

E-mail: matej.velicky@jh-inst.cas.cz; martin.kalbac@jh-inst.cas.cz

Table S1: Elemental concentrations (in atomic %) calculated from high-resolution X-ray photoemission spectra for MoS_2-Ti heterostructure. Layers marked by an asterisk were measured by micro-XPS.

	C	Mo	N	O	S	Ti
Altered 1L UHV	8.9 ± 0.4	10.1 ± 0.1	0.0 ± 0.0	16.1 ± 0.5	17.3 ± 0.3	47.5 ± 0.4
Altered 1L air	28.2 ± 0.3	2.5 ± 0.1	0.6 ± 0.1	44.5 ± 0.2	3.7 ± 0.1	20.5 ± 0.2
Altered 1L*	25.9 ± 1.3	2.5 ± 0.3	1.5 ± 0.7	47.3 ± 1.4	3.5 ± 0.7	19.3 ± 1.0
2L*	33.8 ± 2.6	7.3 ± 0.9	2.9 ± 1.8	24.9 ± 3.1	14.2 ± 1.4	16.9 ± 2.3
3L*	29.0 ± 3.4	14.9 ± 1.4	0.9 ± 0.7	6.7 ± 2.5	33.2 ± 2.3	15.2 ± 1.9

Table S2: Calculation of MoS_2 fraction in individual layers.

Scenario 1			
	MoS_2 content in %		
Measurement region	alt-1L	2L	3L
alt-1L	6		
2L	6	54	
3L	6	78	100
Scenario 2			
	MoS_2 content in %		
Measurement region	alt-1L	2L	3L
alt-1L	6		
2L	15	49	
3L	30	64	100

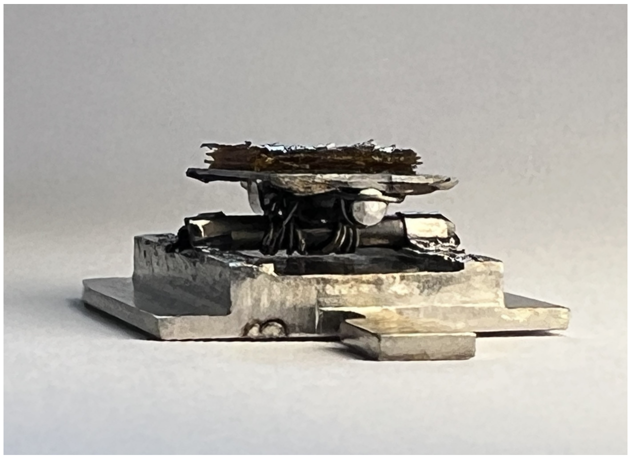

Figure S1: Custom-made rocking sample holder with cleaved MoS_2 crystal on a Kapton tape.

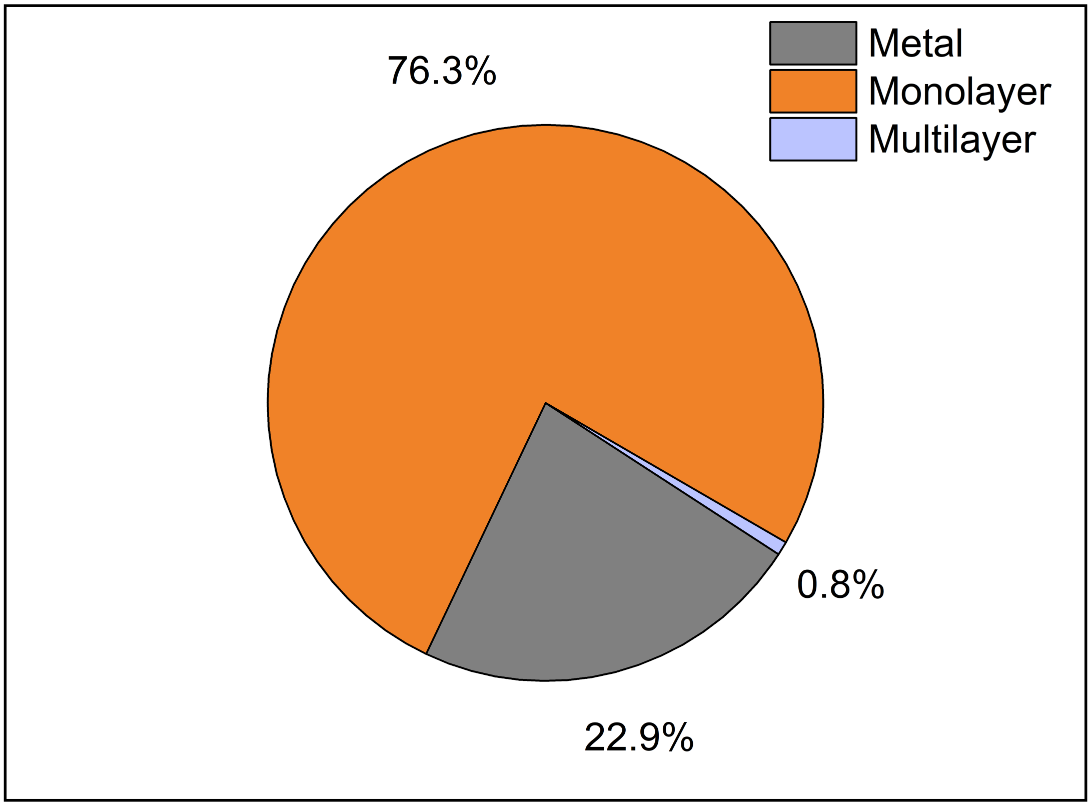

Figure S2: Exfoliation yield expressed as a percentage of the total area within the region marked by the dashed line in Fig. 1(a).

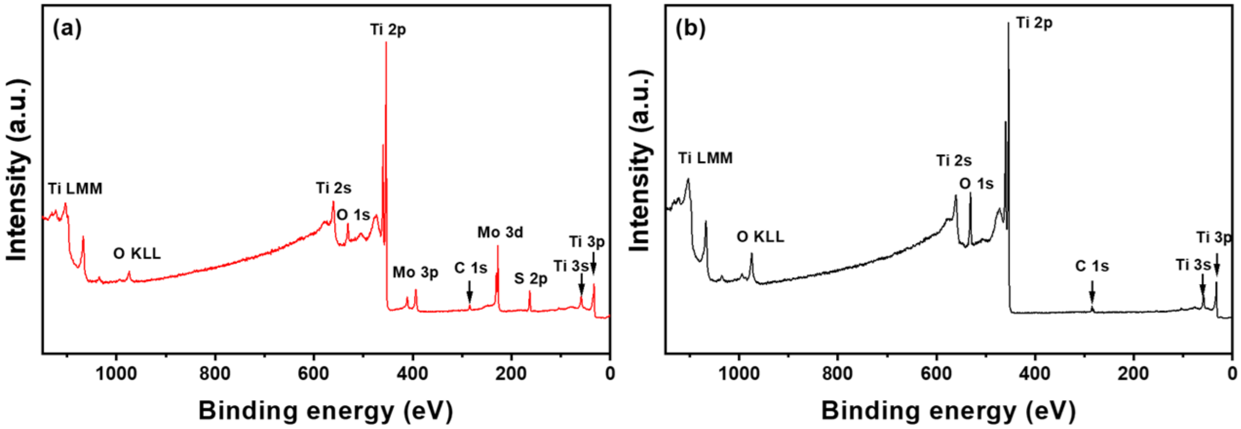

Figure S3: XPS survey spectra from (a) the sample region with the exfoliated layer (marked by a red circle in Fig. 1(a)) and (b) the sample region outside the exfoliated layer (marked by a black circle in Fig. 1(a)).

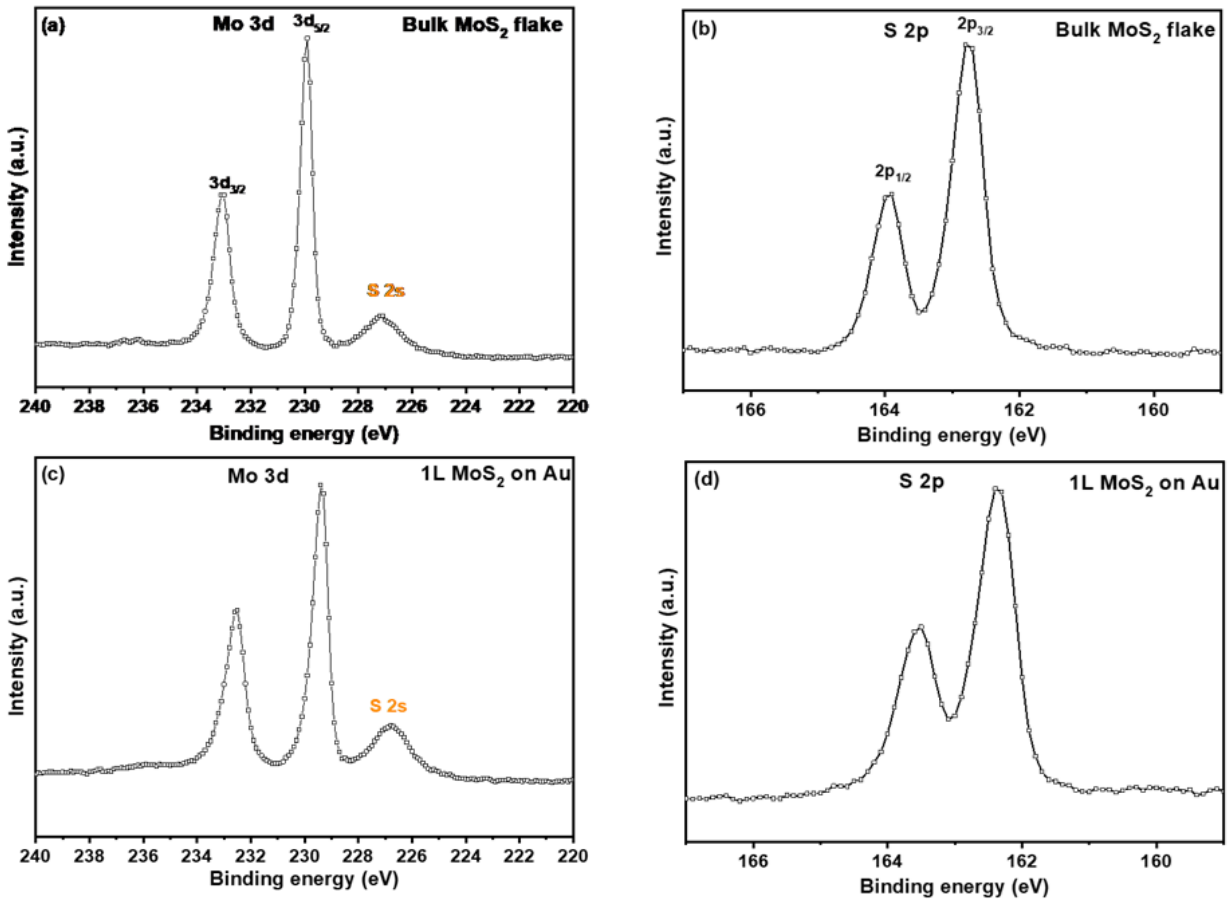

Figure S4: XPS spectra of Mo 3d and S 2p region of bulk flake of MoS_2 (a), (b) and monolayer MoS_2 on Au(111) (c), (d).

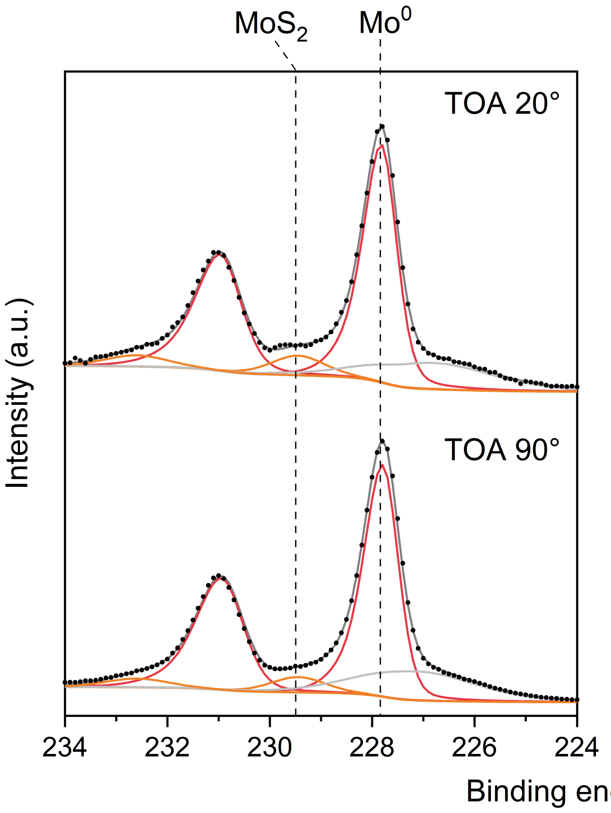

Figure S5: XPS spectra of Mo 3d and S 2p region of exfoliated MoS_2/Ti/SiO_2 measured at different take-off angles (TOA).

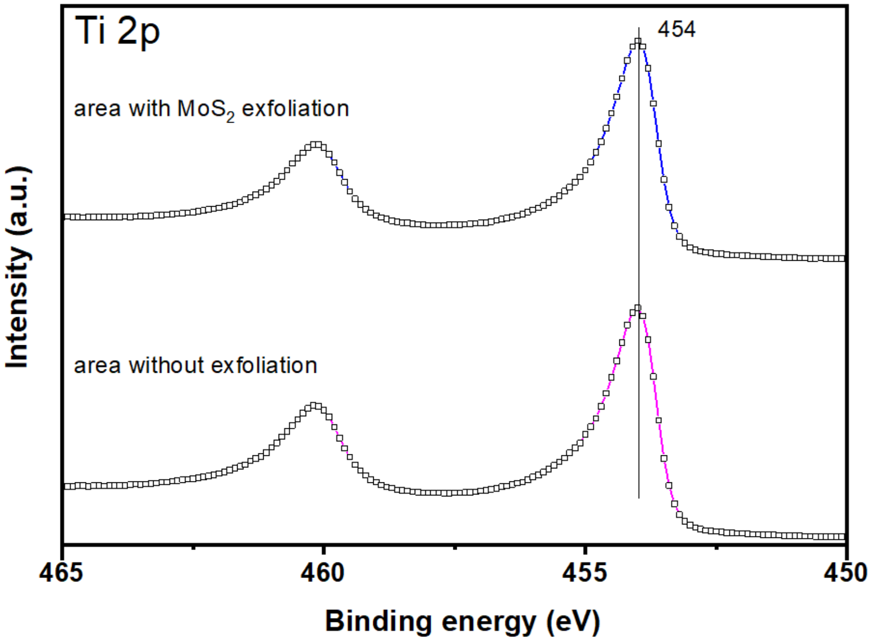

Figure S6: XPS spectra of Ti 2p region from an area with and without exfoliated MoS_2 (take-off angle = 90°).

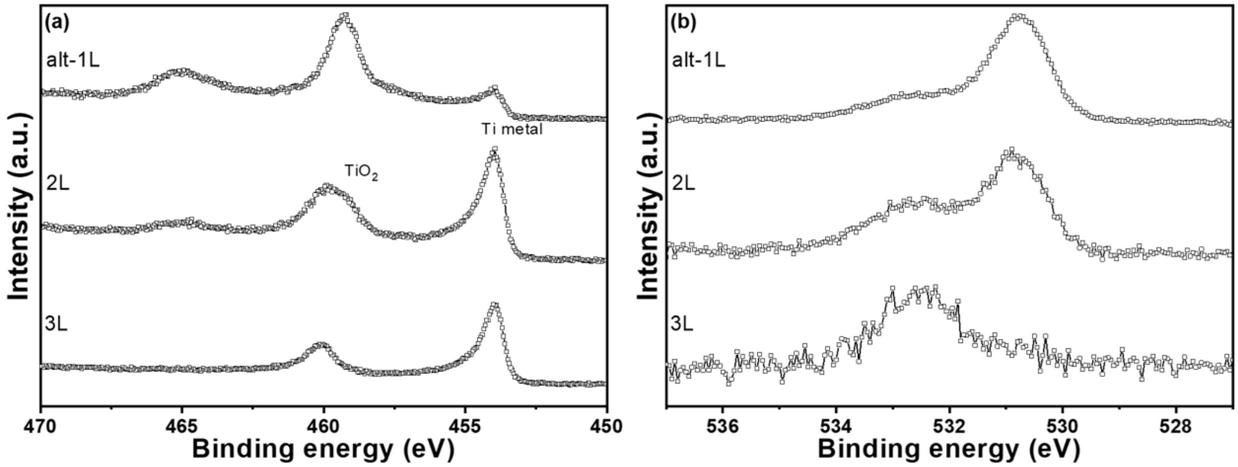

Figure S7: Micro-XPS spectra of Ti 2p (a) and O 1s (b) region of alt-1L, 2L and 3L.

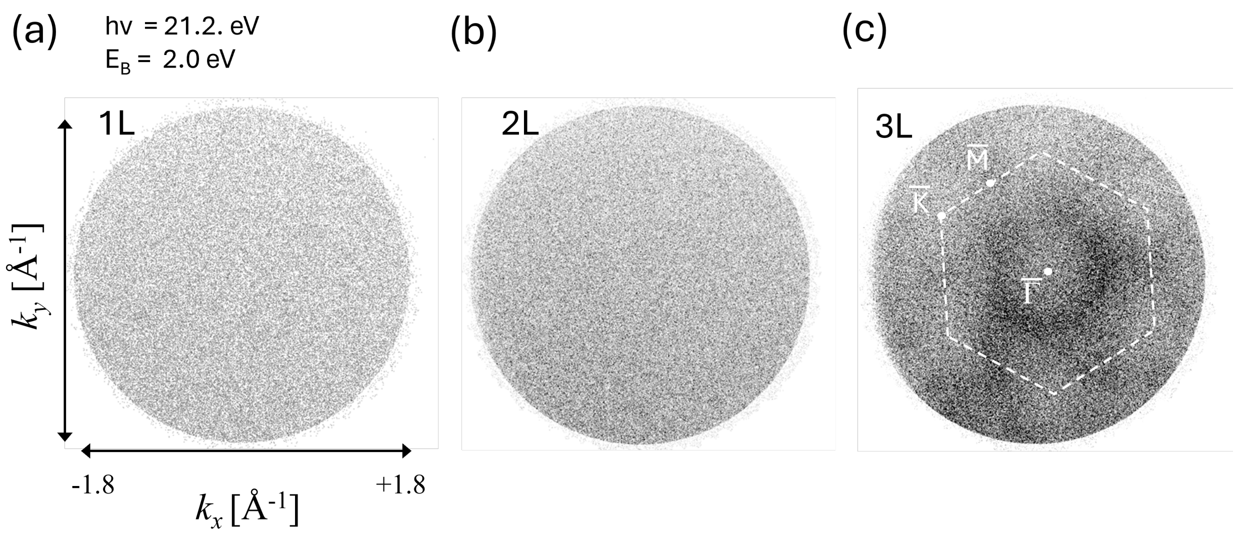

Figure S8: Micro-ARPES data for (a) alt-1L, (b) 2L, and (c) 3L MoS_2 areas on Ti. The images show (k_x,k_y)-dependent photoemission intensities at E_B=2.0 eV.

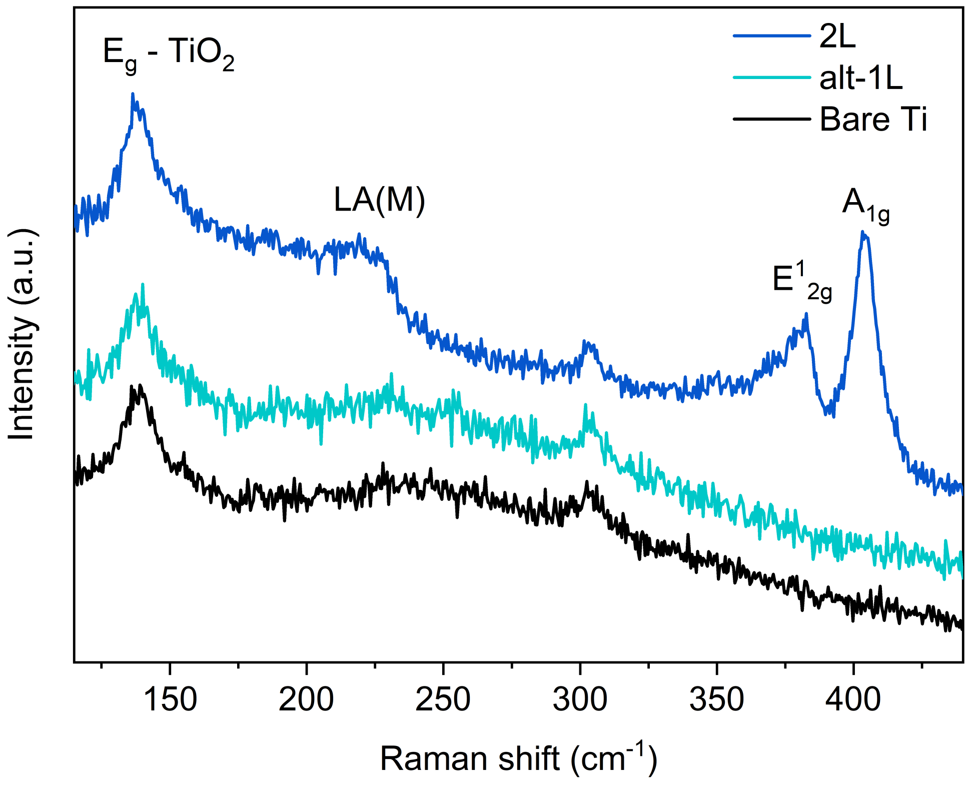

Figure S9: 2L MoS_2 spectrum with the defect peak at approx. 200 cm^{-1}.

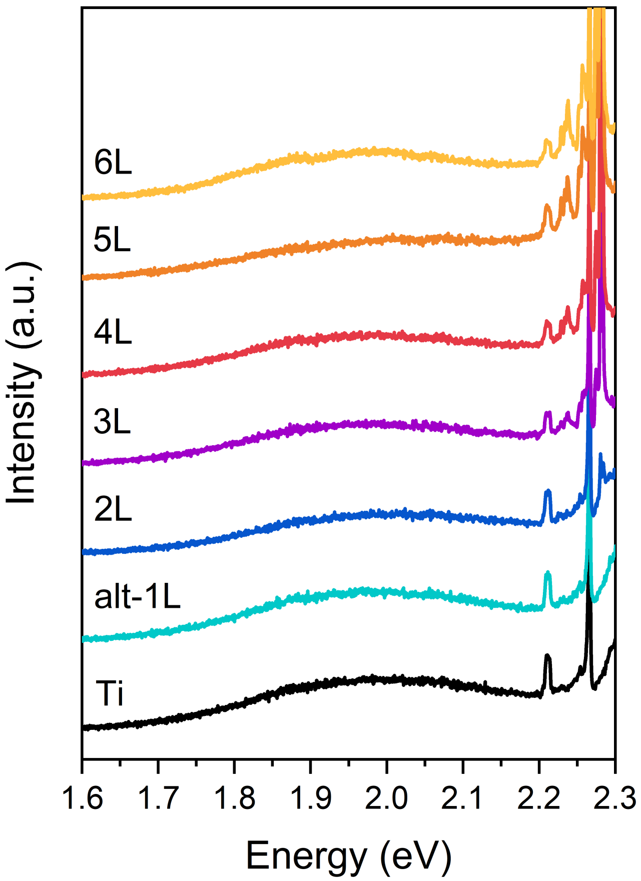

Figure S10: PL spectra of different numbers of MoS_2 layers. No PL emission is observed up to 6L due to the decomposition of the first layer and proximity quenching/band structure changes in thicker layers.

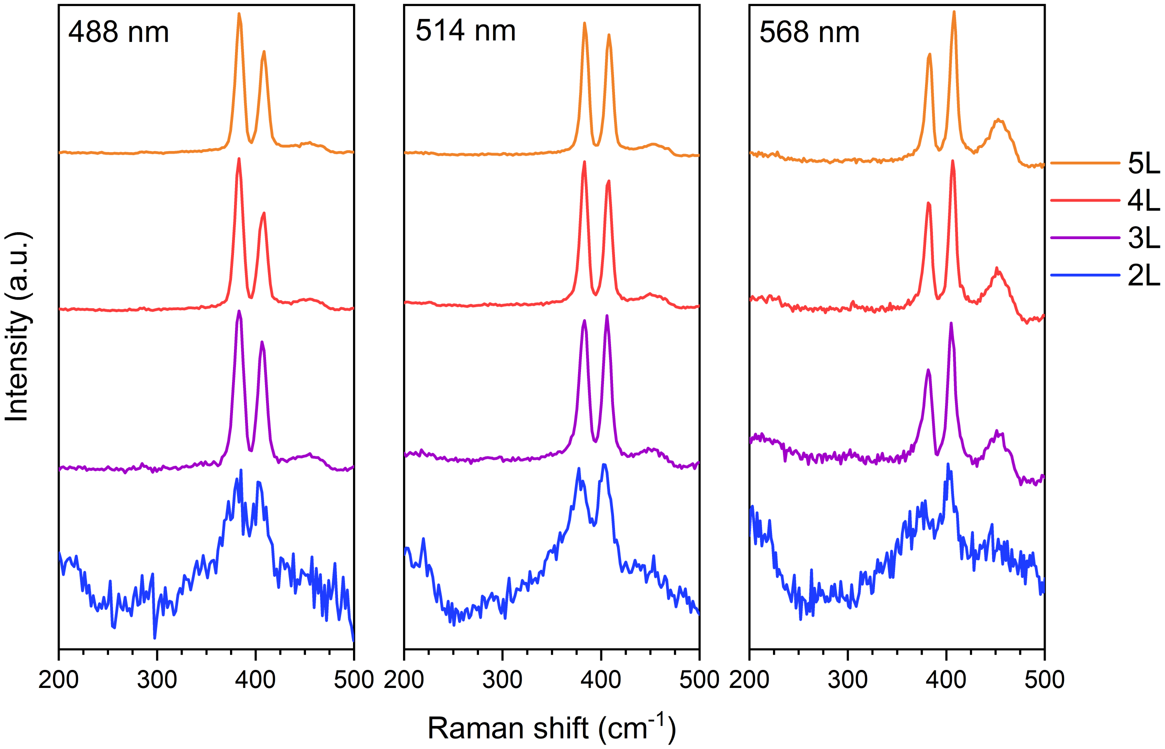

Figure S11: Layer-dependent Raman spectra of MoS_2 on Ti taken with the 488 nm, 514 nm, and 568 nm excitation wavelength.
